# Declining insulin sensitivity is a key pathological contributor to dysglycemia: a longitudinal validation study in the Korean genome and epidemiology study

**DOI:** 10.3389/fendo.2025.1726006

**Published:** 2026-01-05

**Authors:** Doohwa Kim, Jinmi Kim, Myungsoo Im, Soree Ryang, Minsoo Kim, Yeong Jin Kim, In Joo Kim, Young Jin Kim, Hyuk Kang, Stephane T. Chung, Arthur S. Sherman, Sang Soo Kim, Joon Ha

**Affiliations:** 1 Division of Endocrinology and Metabolism, Department of Internal Medicine, Pusan National University Hospital, Busan, Republic of Korea; 2 Biomedical Research Institute, Pusan National University Hospital, Busan, Republic of Korea; 3 Department of Internal Medicine, Pusan National University School of Medicine, Yangsan, Republic of Korea; 4 Department of Biostatistics, Clinical Trial Center, Biomedical Research Institute, Pusan National University Hospital, Busan, Republic of Korea; 5 Center for Global R&D Data Analysis, Division of Data Analysis, Korea Institute of Science and Technology Information (KISTI), Seoul, Republic of Korea; 6 Division of Fundamental Research on Public Agenda, National Institute for Mathematical Sciences, Daejeon, Republic of Korea; 7 Section on Pediatric Diabetes, Obesity, and Metabolism, Diabetes, Endocrinology, and Obesity Branch, National Institute of Diabetes, Digestive and Kidney Diseases, National Institutes of Health, Bethesda, MD, United States; 8 Laboratory of Biological Modeling, National Institute of Diabetes and Digestive and Kidney Diseases, National Institutes of Health, Bethesda, MD, United States; 9 Department of Mathematics, Howard University, Washington DC, United States

**Keywords:** beta-cell function, body mass index, disposition index, insulin resistance, oral glucose tolerance test (OGTT)

## Abstract

**Background:**

The role of worsening insulin resistance (IR) in type 2 diabetes (T2D) progression among lean individuals is not well understood. We examined whether declining insulin sensitivity (IS), independent of beta-cell function (BCF), is associated with diabetes progression.

**Methods:**

In a 10-year longitudinal Korean cohort with normal glucose tolerance (N = 2,810; age 50 ± 8y; BMI 24.1 ± 2.8 kg/m²), participants underwent biannual oral glucose tolerance tests. Longitudinal changes in IS, BCF, BMI, and fat mass were evaluated. Participants were stratified by baseline BCF (low/high), diabetes progression status (non-progressors [Non-p], progressors to prediabetes [PreDM-p], or T2D [T2D-p] over time), and degree of IS decline (large/small).

**Results:**

Both T2D-p and PreDM-p showed greater IS decline than Non-p. At baseline, PreDM-p had IS comparable to T2D-p, but T2D-p declined more steeply. T2D-p and PreDM-p also had lower BCF at baseline, which remained low. Across baseline BCF groups (high/low), greater IS decline was consistently associated with higher risk of progression (all P<0.001). The effect size of IS decline exceeded that of BCF deterioration.

**Conclusions:**

Declining IS, independent of BCF, is associated with progression to T2D in lean Koreans. Preserving IS through lifestyle interventions is crucial for diabetes prevention, particularly in those with low BCF. Insulin resistance, β-cell function, disposition index, oral glucose tolerance test, body mass index.

## 1 Introduction

Failure of the compensatory mechanism of pancreatic beta-cells in response to decreased whole-body insulin action is generally accepted as the most common pathway to type 2 diabetes (T2D) (1–3). The failure of beta-cell function (BCF) and insulin resistance (IR, decreased insulin action), are two major risk factors for progression to T2D (4, 5). In persons with obesity, IR is a main driver of T2D progression and a common interventional target. However, progression to T2D is heterogeneous across ethnic populations. Among East Asian persons, failure of beta-cell response and low BCF at baseline are known to be major causes of T2D (6). However, for designing interventions it is crucial to understand whether and how much longitudinal changes in insulin sensitivity is associated with the development of T2D. Specifically, in Koreans, we examined the contribution of worsening insulin resistance with respect to higher and lower BCF at baseline. If progressors maintain their baseline insulin sensitivity, can T2D be avoided? We address these questions by investigating a longitudinal data set from a Korean cohort. We aimed to 1) investigate insulin sensitivity (IS) and BCF in non-progressors vs. progressors to dysglycemia at baseline and over 10 years; 2) identify whether progressors experienced greater decline in insulin sensitivity than non-progressors over the study period; 3) investigate the independent association of longitudinal changes of IS on progression to T2D within groups with higher and lower BCF at baseline; 4) compare the relative contributions of longitudinal change of IS and BCF to association with dysglycemia over time.

## 2 Materials and methods

### 2.1 Study population

Study data were obtained from the Ansung-Ansan Cohort study, which was part of the Korean Genome Epidemiology study cohorts (KoGES) to identify risk factors for chronic diseases common among Koreans. This is an ongoing prospective, community-based cohort study that contains data from two representative urban and rural communities in Korea. From this cohort, which investigates evidence of major chronic disease longitudinally, we selected subjects who showed no evidence of prediabetes or diabetes at baseline (2001–2002). Data from 2001–2002 represent baseline recruitment, and follow-up data were collected every 2 years (7). The Institutional Review Board of Pusan National University (South Korea) approved the study protocol (IRB No. 2101-007-099). All eligible people ranged in age from 40 to 69 years. In Ansung district community, 5,018 individuals were surveyed with a cluster-sampling method, stratified by age, sex and residential district. In Ansan, 5,012 people were recruited using a random sampling method.

Of 10,030 participants, we excluded those who were diagnosed with diabetes or prediabetes at baseline (n=5,528) (See **Supplementary Figure S1** for details of inclusion and exclusion criteria). Among 4,502 patients who satisfied the normal glucose tolerance (NGT) criteria at baseline and had no history of diabetes related medication use, 7 individuals did not have an oral glucose tolerance test (OGTT) and were excluded (**Supplementary Figure S1**). Additionally, those with no OGTT values at the 10-year follow up were excluded (N = 1,687) (**Supplementary Figure S1**). Finally, 2,810 subjects were eligible for analysis to predict future diabetes or prediabetes. During the 10-year follow-up period, 1,986 participants progressed to prediabetes or diabetes.

### 2.2 Measurement of anthropometric and biochemical parameters

All participants had a medical history taken, a physical examination, and hematologic and biochemical tests performed. Body mass index (BMI) was calculated by measuring height and weight to the nearest 0.1cm and 0.1kg, respectively. Blood pressure was measured using standard methods. Biochemical measurements such as fasting plasma glucose (FPG), insulin, total cholesterol, triglycerides, high-density lipoprotein (HDL) were obtained after at least 8 hours overnight fasting and were measured enzymatically using a 747 Chemistry Analyzer (Hitachi, Tokyo, Japan). Plasma glucose concentrations were measured by the hexokinase method. HbA1c levels were measured by high-performance liquid chromatography (Variant II; BioRad Laboratories, Hercules, CA, USA). Plasma insulin concentrations were measured by radioimmunoassay (LINCO kit, St Charles, MO, USA). Each participant underwent a two-hour 75g oral glucose tolerance test and had medical history and blood taken at the baseline and every biannual visit. After overnight fasting, plasma glucose (PG) and insulin (PI) was measured at the 0-, 60- and 120-min time points of the OGTT.

### 2.3 Definitions of normal glucose tolerance, prediabetes and diabetes

NGT was defined as fasting plasma glucose (FPG) concentration <100 mg/dL (5.6 mmol/L), 2-hour PG concentration <140 mg/dL (7.8 mmol/L), and HbA1c <5.7%. Prediabetes was defined by at least one of the following criteria: (1) FPG ≥ 100 mg/dL (5.6 mmol/L) and <126 mg/dL (7.0 mmol/L); or (2) HbA1c of 5.7–6.4%; or (3) 2-hour PG of 140–199 mg/dl (7.8–11.1 mmol/L). Diabetes was defined when at least one or more of the following conditions were satisfied: (1) FPG ≥ 126mg/dl (7.0 mmol/L); (2) HbA1c ≥ 6.5%; (3) 2-hour PG ≥ 200 mg/dl (11.1 mmol/L), or (4) current treatment with insulin or oral antidiabetic drugs as referenced by the 2024 American Diabetes Association standards (8). Dysglycemia was defined as prediabetes or diabetes.

### 2.4 Definitions of mathematical model-derived parameters

The previously validated Insulin Sensitivity and Secretion (ISS) model was used with Matlab (v.9.5.0 (R2021b), Natick, MA: The MathWorks Inc) to estimate insulin sensitivity (model-derived sensitivity of insulin, mSI), β-cell function (model-derived BCF, mBCF) and model-derived DI (mDI) over time (9). See the codes and equations in the **Supplementary Material** of the reference paper at https://doi.org/10.6084/m9.figshare.23535612.

### 2.5 Definitions of metabolic parameters

The cohort was divided into two groups, low and high BCF, by the median of mBCF at baseline. Decrease in insulin sensitivity (DIS) was calculated by subtracting mSI at year 10 from mSI at baseline. Decrease in mBCF (DBCF) was calculated by the subtracting mBCF at year 10 from mBCF at baseline. Positive values of DIS and DBCF mean decrease of the respective values. The insulinogenic index (IGI60) was also used to assess BCF and was calculated as the ratio of the incremental change of insulin and glucose from 0 to 60 minutes of the OGTT (10). The Matsuda index assessing insulin sensitivity was calculated for two-hour OGTTs as (11).


$$\frac{10000}{\sqrt{\bar{I}\bar{G}I_{o}G_{0}}},$$

where $I_{0}$ and $G_{0}$ are the fasting insulin (μU/mL) and glucose (mg/dl) and $\bar{I}$ and $\bar{G}$ are the average insulin and glucose over the OGTT (0, 60, 120 min) calculated as AUC/120 min by the trapezoidal rule.

Using the same definitions and units, HOMA IS, the reciprocal of HOMA IR, was calculated as (12).


$$\frac{405}{I_{0}G_{0}},$$

and HOMA Beta was calculated as (12).


$$\frac{360I_{0}}{G_{0}-63}.$$

mSI was multiplied by mBCF to determine the model derived disposition index (mDI) (13). IGI60 was multiplied by the Matsuda index to determine the oral disposition index (oDI) (11).

### 2.6 Statistical analysis

Continuous variables are presented as mean and standard deviation (SD). Categorical variables are presented as frequency and percentage. Comparisons of two groups were done by two-sample t-test for continuous data and chi-squared test for categorical data. Comparisons among three groups were done by one-way ANOVA for continuous data and the chi-squared test for categorical data. The *post hoc* test was done by the Bonferroni method. To test whether decreased insulin sensitivity are associated with the progression to dysglycemia over the study duration, we conducted univariate and multivariate analyses using a logistic regression model with progression to dysglycemia at any time during the study as the dependent variable, and clinical and metabolic variables as independent variables. Both the unstandardized and standardized coefficients were adjusted for age, sex, BMI, insulin sensitivity, and beta-cell function at baseline. We carried out Little’s MCAR (Missing Completely at Random) test to check whether the participants removed because of incomplete data (section 2.1) differed from those who remained and found no clinically meaningful differences. Statistical analysis was performed using R version 4.3.1 (R Core Team, 2023, http://cran.r-project.org) and additional packages (tableone, ggpubr, ggplot2, pROC, MCAR). A p-value <0.05 was considered statistically significant.

## 3 Results

### 3.1 Baseline characteristics

A total of 2,810 participants who were NGT at baseline were included in this analysis. Of those, 824 maintained NGT (non-progressors, Non-p group), and 1,803 progressed to prediabetes (PreDM-p group) and 183 progressed to diabetes (T2D-p group) during the 10-year follow-up period. **Table 1** shows the anthropometric measurements of Non-p and PreDM-p, and T2D-p at baseline. Age, BMI, fat mass, waist circumference, blood pressure, total cholesterol, and triglyceride were all lower in Non-p, compared to PreDM-p and T2D-p (All *P* < 0.001). Glucose at t = 0, 60, and 120 min of the OGTT and HbA1c were lower in Non-p than PreDM-p or T2D-p (All *P* < 0.001).

**Table 1:** Anthropometrics of study participants at baseline and Follow-up (N = 2,810).

Characteristics	Overall (N = 2,810)	Non-p (N = 824)	PreDM-p (N = 1,803)	T2D-p (N = 183)	*P* value
Age, years	50.0 (8.1)	48.6 (7.6)	50.5 (8.2)^†^	51.3 (8.3)^†^	<0.001
Sex (male), n (%)	1,304 (46.4)	364 (44.2)	832 (46)^†^	108 (59.0)^†‡^	0.001
BMI, kg/m^2^	24.1 (2.9)	23.6 (2.8)	24.4 (2.9)^†^	24.2 (3.1)^†^	<0.001
Body fat mass, kg	16.3 (5.0)	15.3 (4.7)	16.7 (5.02) ^†^	16.3 (4.9)	<0.001
Body fat free mass, kg	46.3 (8.3)	46.0 (8.4)	46.2 (8.3)	46.8 (8.4)	0.523
Waist circumference, cm	81.0 (8.3)	79.2 (7.8)	81.7 (8.4)^†^	82.4 (8.3)^†^	<0.001
SBP, mmHg	114.0 (16.7)	111.3 (15.7)	115.0 (17.0)^†^	116.9 (16.9)^†^	<0.001
DBP, mmHg	73.8 (11.0)	72.4 (10.5)	74.2 (11.2)^†^	76.0 (10.9)^†^	<0.001
Laboratory findings					
HbA1c, %	5.34 (0.23)	5.25 (0.23)	5.37 (0.23)^†^	5.40 (0.19)^†^	<0.001
Creatinine, mg/dL	0.83 (0.17)	0.83 (0.17)	0.83 (0.18)	0.85 (0.19)	0.191
Total cholesterol, mg/dL	184.9 (32.6)	179.3 (29.7)	187.1 (33.6)^†^	188.7 (31.8)^†^	<0.001
LDL, mg/dL	113.4 (30.5)	110.4 (27.2)	114.7 (31.8)^†^	113.1 (31.4)	0.002
HDL, mg/dL	45.2 (9.9)	45.9 (9.8)	45.0 (10.0)	43.8(10.0)^†^	0.016
Triglyceride, mg/dL	131.9 (84.5)	115.1 (64.7)	136.8 (90.0)^†^	158.7 (93.9)^†‡^	<0.001
OGTT measures					
Glucose (0 min), mg/dl	80.5 (6.8)	78.7 (6.3)	81.1 (6.7)^†^	82.3 (7.9)^†^	<0.001
Glucose (60 min), mg/dl	124.9 (32.6)	112.5 (29.3)	128.3 (31.7)^†^	147.5 (34.8)^†‡^	<0.001
Glucose (120 min), mg/dl	101.5 (20.0)	95.7 (19.0)	103.4 (20.0)^†^	108.4 (18.8)^†‡^	<0.001
Insulin (0 min), µU/ml	7.37 (4.99)	6.88 (3.51)	7.58 (5.47)^†^	7.49 (5.52)	<0.001
Insulin (60 min), µU/ml	30.85 (30.69)	28.16 (25.55)	25.34 (23.34)^†^	32.29 (33.44)	0.004
Insulin (120 min), µU/ml	24.31 (22.02)	21.70 (18.67)	25.34 (23.34)^†^	25.97 (21.72)^†^	<0.001
Metabolic estimates					
HOMA-IR	1.47 (1.01)	1.34 (0.68)	1.52 (1.11)^†^	1.54 (1.22)	<0.001
HOMA-β	178.76 (157.89)	187.88 (155.36)	175.88 (161.08)	166.08 (134.26)	0.082
Matsuda index	12.8 (9.5)	14.1 (3.2)	12.3 (9.0)^†^	11.8 (7.8)^†^	<0.001
IGI	1.0 (3.1)	1.1 (0.5)	1.3 (0.5)	1.3 (0.6)^†‡^	<0.001
mSI	1.32 (0.52)	1.39 (0.46)	1.30 (0.53)^†^	1.28 (0.60)	<0.001
mBCF	0.38 (0.26)	0.44 (0.26)	0.36 (0.25)^†^	0.29 (0.28)^†‡^	<0.001
mDI	3.03 (1.66)	3.76 (1.68)	2.79 (1.55)^†^	2.02 (1.37)^†‡^	<0.001

Variables are showed as number (%), mean (SD), BMI, body mass index; SBP, systolic blood pressure; DBP, diastolic blood pressure; HbA1c, glycated hemoglobin; LDL, low density lipoprotein; HDL, high density lipoprotein; HOMA-IR, homeostatic Model Assessment for Insulin Resistance; HOMA-β, homeostatic Model Assessment for beta cell function; IGI, insulinogenic index; OGTT, oral tolerance test; mSI, model-derived insulin of sensitivity; mBCF, model-derived beta-cell function; mDI, model-derived disposition index. †P < 0.05 vs. Non-p, ‡P < 0.05 vs. PDM-p.

### 3.2 Longitudinal changes in insulin sensitivity and beta-cell function

**Figure 1:**
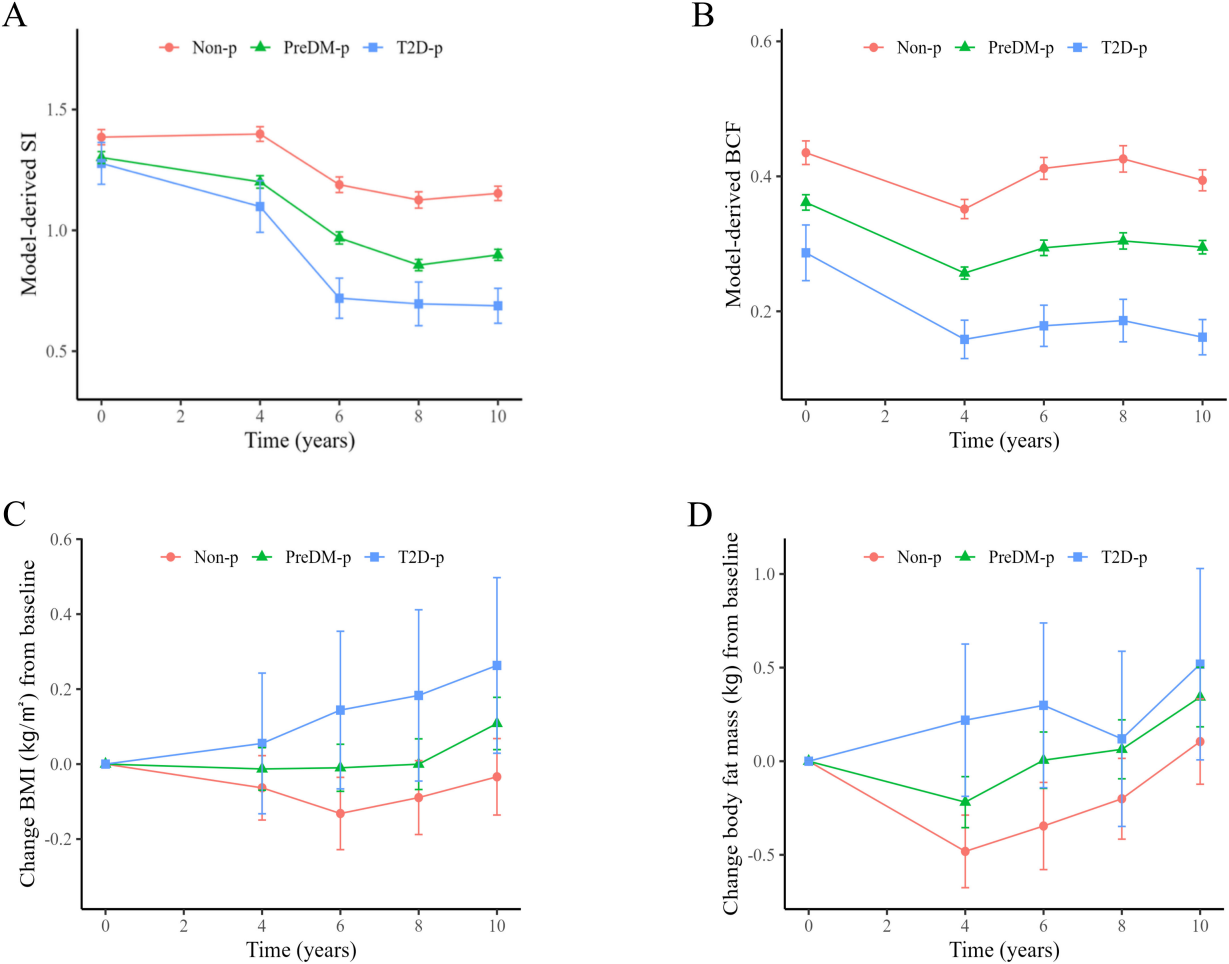
Longitudinal changes of insulin sensitivity (IS), beta-cell function (BCF), body mass index (BMI) and body fat mass. **(A)** The longitudinal changes of model-derived sensitivity of insulin (mSI). At baseline, mSI is higher in Non-p than the two progressor groups, but there is no difference in mSI between PreDM-p and T2D-p. Over time, mSI is decreased in all groups. T2D-p exhibit the most worsened insulin resistance (IR), PreDM-p intermediate, and Non-p the least over time. **(B)** All the three groups are different in model-derived beta-cell function (mBCF) at baseline and decreases, compared to their respective baseline. T2DM-p show the largest reduction from baseline, PreDM-p intermediate, and Non-p the least. **(C)** The longitudinal changes of BMI from baseline. BMIs of both progressors tend to keep increase and become significantly different from the baseline at year 10. In contrast, BMI of Non-p does not increase. **(D)** The longitudinal changes of body fat mass from baseline. Body fat mass in the two progressor groups increase over time but does not increase in the Non-p. ^*^P < 0.05 vs. baseline. See Supplemental **Supplementary Tables S1, S2** for detailed statistics.

Insulin sensitivity determined by the ISS model (mSI) was higher in Non-p than T2D-p and PreDM-p at baseline (**Figure 1**, **Table 1**). There was no significant difference in mSI between PreDM-p and T2D-p at baseline. During the 10-year follow-up period, mSI decreased in Non-p and the two progressor groups. mSI at the last follow-up decreased by 46.2% compared to the baseline in T2D-p (from 1.28 to 0.69 on average), 31.0% in PreDM-p (from 1.30 to 0.90), and 16.8% (from 1.39 to 1.15) in Non-p (**Figure 1A**) (see **Supplementary Table S1** for details of statistics).

Model-derived beta cell function (mBCF) at baseline was different in the three progression groups: high, intermediate, and low across Non-p, PreDM-p and T2D-p. mBCF at the last follow-up decreased by 43.6% compared to baseline in T2D-p (from 0.29 to 0.16), 18.4% in PreDM-p (from 0.36 to 0.30), and 9.4% in Non-p (from 0.44 to 0.39) (**Figure 1B**). OGTT-derived markers of IGI and Matsuda index showed the same patterns at baseline and over the longitudinal study (see **Supplementary Figure S3**, **Supplementary Table S3** for details of the statistics). Beta-cell function relative to insulin sensitivity decreased in all three groups but declined most in the T2D-p group, followed by the PreDM-p group (66.4%, 41.4%, 22.2%). The model-derived disposition index (mDI) shows the pattern more clearly than the oral disposition index (oDI) (**Supplementary Figure S4**).

### 3.3 Longitudinal increments of body mass index and fat mass

Longitudinal changes of BMI and fat mass are shown in **Figures 1C, D**. Both BMI and fat mass of the two progressor groups increased at the last follow-up, compared to the respective baseline values (All *P* < 0.05), while BMI and fat mass of Non-p did not change (*P* = 0.515 and *P* = 0.367, respectively) (see **Supplementary Table S1** for details of statistics).

### 3.4 Effect of longitudinal change in insulin sensitivity on future dysglycemia when baseline beta-cell function is similar

To further examine the association of longitudinal changes in insulin sensitivity on future dysglycemia, we compared two groups with low and high BCF defined in section in order to neutralize the influence of baseline BCF. Each group was split into two sub-groups, large and small decrease in insulin sensitivity (large DIS; small DIS) by the median of changes of mSI. Within the low BCF group, the large DIS group had higher incidence of T2D-p and PreDM-p than the small DIS group (6.4% vs. 2.6%, *P* = 0.001; 50.9% vs. 33.9%, *P* < 0.001, respectively). The same pattern was observed within the high BCF group; large and small DIS (2.6% vs. 1.3%, *P* = 0.08, 38.3% vs. 27.1%, *P* < 0.001, respectively) (**Table 2**). The cohort was also divided into low and high insulin sensitivity at baseline. Within each group, decreased beta-cell function was associated with higher incidence of future dysglycemia (**Supplementary Table 6**). The same pattern was observed when the analyses were stratified by tertiles and quartiles of baseline BCF (not shown).

**Table 2:** Incidence of diabetes and pre-diabetes in groups of high and low beta-cell function, subdivided by small and large decrease in insulin sensitivity (DIS).

	Low Beta-cell function			High Beta-cell function		
	Large DIS (N = 703)	Small DIS (N = 702)	*P* value	Large DIS (N = 703)	Small DIS (N = 702)	*P* value
T2D-p	45 (6.4%)	18 (2.6%)	0.001	18 (2.6%)	9 (1.3%)	0.081
PreDM-p	358 (50.9%)	237 (33.8%)	<0.001	269 (38.3%)	190 (27.1%)	<0.001
Non-p	300 (42.7%)	447 (63.7%)	<0.001	416 (59.3%)	503 (71.7%)	<0.001

DIS, Decreased insulin sensitivity = insulin sensitivity at year 0 - insulin sensitivity at year 10

### 3.5 Relative associations of longitudinal changes in insulin sensitivity and beta-cell function to future dysglycemia

To assess the relative associations of longitudinal changes in insulin sensitivity and beta-cell function to progression to dysglycemia, logistic regression was carried out and standardized regression coefficients were calculated by scaling and centering variables expressed in different units (**Table 3**). Both decreased insulin sensitivity (DIS) and decreased beta-cell function (DBCF) were significantly associated with progression to prediabetes and T2D (all *P* < 0.001, **Table 3**) according to both the unstandardized and standardized analyses. The effect sizes (β) of DIS were consistently larger than those of the DBCF in predicting progression to prediabetes and T2D across all models. Model 1 was unadjusted (crude), including only DIS and DBCF; Model 2 was adjusted for age, sex, and BMI; and Model 3 was further adjusted for baseline insulin sensitivity and beta-cell function.

**Table 3:** Longitudinal decrease in insulin sensitivity (DIS) and beta-cell function (DBCF) by multiple logistic regression.

Outcome	Parameter	Model 1				Model 2				Model 3			
		Unstandardized	Standardized	p-value	Ratio of	Unstandardized	Standardized	p-value	Ratio of	Unstandardized	Standardized	p-value	Ratio of
		β (95% CI)	β (95% CI)		Standardized coefficients	β (95% CI)	β (95% CI)		Standardized coefficients	β (95% CI)	β (95% CI)		Standardized coefficients
Progression to preDM	DIS	0.36 (0.22, 0.50)	0.22 (0.14, 0.30)	<0.001	1.833	0.37 (0.23, 0.51)	0.22 (0.14, 0.31)	<0.001	2.200	0.56 [0.40, 0.72]	0.34 [0.24, 0.44]	<0.001	2.429
	DBCF	0.43 (0.13, 0.74)	0.12 (0.04, 0.20)	0.006	Ref.	0.36 (0.05, 0.67)	0.10 (0.01, 0.18)	0.024	Ref.	0.51 [0.18, 0.84]	0.14 [0.05, 0.23]	0.003	Ref.
Progression to DM	DIS	0.93 (0.66, 1.20)	0.57 (0.40, 0.73)	<0.001	1.295	0.96 (0.69, 1.23)	0.59 (0.42, 0.75)	<0.001	1.372	1.29 [0.97, 1.62]	0.79 [0.59, 0.99]	<0.001	1.145
	DBCF	1.59 (1.04, 2.13)	0.44 (0.29, 0.59)	<0.001	Ref.	1.56 (1.00, 2.11)	0.43 (0.28, 0.58)	<0.001	Ref.	2.49 [1.82, 3.17]	0.69 [0.50, 0.87]	<0.001	Ref.

The ratio of standardized coefficients was used to compare the relative strength of direct effects; Ref., DBCF as reference

Model 1: DIS + DBCF.

Model 2: Model 1 + sex, age, BMI.

Model 3: Model 2 + baseline si and sigma (categorical: high vs. low by median).

β indicates the regression coefficient estimated from the logistic regression model.

CI, confidence interval; DIS, decreased insulin sensitivity; DBCF, decreased beta-cell function.

Logistic regression analyses were performed using three models.

Model 1: included decreased insulin sensitivity (DIS) and decreased beta-cell function (DBCF).

Model 2: additionally adjusted for sex, age, and body mass index (BMI).

Model 3: further included baseline si and sigma (categorical: high vs. low by median).

### 3.6 Independent association of longitudinal change in insulin sensitivity with future dysglycemia

To confirm the independent association of DIS with progression to dysglycemia, multiple logistic regression analyses were performed with adjustment for baseline insulin sensitivity, baseline beta-cell function, and longitudinal changes in beta-cell function (**Supplementary Table S5**). The odds ratios (OR) of predicting progression to PreDM or T2D by DIS remained significant after adding the effects of age, sex, BMI, baseline IS and BCF, and DBCF.

## 4 Discussion

We have shown that T2D and PreDM progressors had greater decline in insulin sensitivity than non-progressors over a 10-year longitudinal cohort study. T2D and PreDM progressors also had lower BCF than the non-progressors at baseline and all follow-ups. Although the population studied was relatively lean, BMI and fat mass increased in the two progressor groups and were associated with declining insulin sensitivity. Worsening IR was associated with risk of future diabetes, independent of baseline BCF and IS and decline in BCF over time and contributed more based on the size of the standardized coefficients to the progression to prediabetes and T2D than DBCF over time. Thus, low BCF was a risk factor for T2D, as expected for an East Asian cohort, but the likelihood of progression, irrespective of high or low BCF, was strongly associated with reductions in insulin sensitivity. Preservation of insulin sensitivity is therefore a priority, especially for individuals with low BCF because they have limited ability to mount a compensatory response to insulin resistance.

### 4.1 Longitudinal changes of insulin sensitivity, beta-cell function, body mass index and fat

At baseline, IS was modestly larger in non-progressors than in PreDM-p and T2D-p (**Figure 1A**, **Table 1**). Over time, IS decreased in all three groups but the magnitude of decline was significantly greater in the progressor groups (Non-p, 18.4%; PreDM-p, 30.7%; T2D-p, 46.5%; See **Supplementary Table S1** for details of statistics). Increased BMI and fat mass over time correlated with declining insulin sensitivity in the two progressor groups.

At baseline, BCF was 18% lower in PreDM-p and 34% lower in T2D-p compared to Non-p (All *P* < 0.001). These significant baseline differences are important for explaining the differences in likelihood of progression to T2D. Furthermore, decline in BCF from baseline to the last follow up was markedly different across the three groups, 9.3% vs. 19.4% vs. 44.8%, which was also associated with T2D risk.

The findings of the current study align with those of (6), which analyzed the same cohort data with different measures of IS and BCF. That study found as we did that 1) low baseline BCF was an important predictor of T2D progression and 2) IS declined more in progressors than non-progressors. That study (6), however, emphasized the role of low baseline BCF compared to that of baseline insulin sensitivity, with failure of beta-cell compensation as the primary pathophysiology. In contrast, we highlight that the contributions to future T2D of longitudinal declines in insulin sensitivity were more associated with the progression to prediabetes and T2D than longitudinal decreases in beta-cell function. Indeed, there is no need for beta-cell compensation in the absence of insulin resistance. In this population, maintaining insulin sensitivity reduces stress on the beta cells and helps to preserve beta-cell function. The prevalent low BCF was adequate for normoglycemia before insulin resistance developed.

Our results also align with a recent study of African immigrants living in the US (14). That study reported that about half of those with T2D had obesity and severe insulin resistance, and half were lean with relatively mild insulin resistance. The lean subgroup with T2D had low BCF comparable to, and IS significantly lower than, an adjacent subset of those with NGT or PreDM. Although cross-sectional, that study is suggestive that a subset of lean people with low baseline BCF progress to T2D through moderate reduction of IS, similar to the KoGES cohort.

### 4.2 Effect of longitudinal change in insulin sensitivity on progression to dysglycemia over time

To answer the initial question, “what if the progressors maintain their IS at baseline levels?”, we have investigated the association of worsening IR on future dysglycemia. The whole group was divided into two subgroups, low and high BCF, by the median value at baseline. Within each BCF group, the subgroup with larger decrease in IS (DIS) had significantly higher probability to develop PreDM and T2D (**Table 2**). This finding supports the hypothesis that preserving insulin sensitivity may limit progression to dysglycemia. By longitudinally simulating a mathematical model of diabetes progression, we confirmed that worsening insulin resistance leads to progression to T2D even when beta-cell function at baseline is equal (**Supplementary Figure 2**) (15). In addition, the subgroup with large DIS and low baseline BCF had the highest incidence of future prediabetes and T2D (**Table 2**). Thus, the combination of worsening IR over time and low BCF at baseline confers the highest risk of future dysglycemia.

### 4.3 Clinical implications

As IR is a risk factor that can be managed by lifestyle intervention, the current findings support intervention in lean people who are at high risk assessed based on low BCF. Those people should be treated by intensive lifestyle interventions at an early stage of dysglycemia. This is supported by a retrospective study of Koreans with average BMI 24.1 (kg/m^2^), which showed that weight loss was correlated with remission of newly diagnosed type 2 diabetes patients (16).

### 4.4 Strengths and limitations

A large, well-planned observational untreated cohort in a single ethnic population was used to determine the association of longitudinal change of IR in future dysglycemia. We determined that worsening IR is important in this East Asian population with a relatively lower BMI (~ 24 kg/m^2^) compared to Western populations. Taken together with results from African immigrant populations, the current findings expand our knowledge of the pathogenesis of diabetes (14). However, the current study is observational, not interventional. A large scale interventional study is needed to validate interventional guidelines for lean individuals with low BCF.

## 5 Conclusion

Worsening IR was a key pathological contributor to the development of diabetes, even in lean people, where relatively poor BCF was known to be a major mechanism in the development of diabetes. Maintaining insulin sensitivity through lifestyle modification may be an easier way to prevent diabetes.

## Data Availability

The data analyzed in this study is subject to the following licenses/restrictions: Data will be made available upon reasonable request. Requests to access these datasets should be directed to drsskim@pusan.ac.kr.

## Author disclaimer

The contributions of the NIH authors are considered Works of the United States Government. The findings and conclusions presented in this paper are those of the authors and do not necessarily reflect the views of the NIH or the US Department of Health and Human Services.
