# Supplementary material for: Declining insulin sensitivity is a key pathological contributor to dysglycemia: a longitudinal validation study in the Korean genome and epidemiology study
Source: Front Endocrinol (Lausanne). 2026 Jan 5;16:1726006. doi: 10.3389/fendo.2025.1726006 (PMC12812719; doi:10.3389/fendo.2025.1726006)
Supplement: Supplementary file 1 [file DataSheet1.pdf]

Supplementary Material – Tables, Figures, and Legends

Kim et. al,

Declining insulin sensitivity is a key pathological contributor to dysglycemia: A Longitudinal Validation Study in the Korean Genome and Epidemiology Study (KoGES)

Supplemental Table S1. Longitudinal changes of beta-cell function and insulin sensitivity (Details of Fig. 1A and B)

			Raw data					Change from baseline			
						Comparison between groups				Comparison between groups	
	Subgroup	Time (year)	N	Mean (SD)	Percentage change	p-value†	p-value‡	Mean difference (95%CI)	p-value ¶	p-value†	p-value‡
mSI	Non-p	0	824	1.39 (0.46)	Ref.			Ref.			
		4	742	1.40 (0.42)	1.1%			0.02 (-0.03,0.06)	0.465		
		6	722	1.19 (0.44)	-13.7%			-0.19 (-0.23,-0.15)	<0.001		
		8	745	1.13 (0.47)	-18.1%			-0.25 (-0.29,-0.21)	<0.001		
		10	824	1.15 (0.43)	-16.8%			-0.23 (-0.27,-0.20)	<0.001		
	PreDM-p	0	1,803	1.30 (0.53)	Ref.	<0.001		Ref.			
		4	1,681	1.20 (0.54)	-7.6%	<0.001		-0.10 (-0.13,-0.07)	<0.001	<0.001	
		6	1,624	0.97 (0.52)	-25.4%	<0.001		-0.33 (-0.36,-0.30)	<0.001	<0.001	
		8	1,704	0.86 (0.49)	-34.2%	<0.001		-0.44 (-0.47,-0.41)	<0.001	<0.001	
		10	1,803	0.90 (0.50)	-31.0%	<0.001		-0.40 (-0.43,-0.37)	<0.001	<0.001	
	T2D-p	0	183	1.28 (0.60)	Ref.	0.022	0.600	Ref.			
		4	171	1.10 (0.71)	-14.9%	<0.001	0.070	-0.19 (-0.32,-0.07)	0.003	0.003	0.160

			Raw data					Change from baseline			
						Comparison between groups				Comparison between groups	
	Subgroup	Time (year)	N	Mean (SD)	Percentage change	p-value†	p-value‡	Mean difference (95%CI)	p-value ¶	p-value†	p-value‡
		6	169	0.72 (0.55)	-43.9%	<0.001	<0.001	-0.56 (-0.67,-0.46)	<0.001	<0.001	<0.001
		8	164	0.70 (0.59)	-46.5%	<0.001	<0.001	-0.61 (-0.72,-0.49)	<0.001	<0.001	0.008
		10	183	0.69 (0.50)	-46.2%	<0.001	<0.001	-0.59 (-0.69,-0.49)	<0.001	<0.001	<0.001
mBCF	Non-p	0	824	0.44 (0.26)	Ref.			Ref.			
		4	742	0.35 (0.20)	-19.5%			-0.08 (-0.11,-0.06)	<0.001		
		6	722	0.41 (0.22)	-7.5%			-0.03 (-0.05,-0.01)	0.003		
		8	745	0.43 (0.27)	-3.7%			-0.02 (-0.04,0.01)	0.154		
		10	824	0.39 (0.23)	-9.4%			-0.04 (-0.06,-0.02)	<0.001		
	PreDM-p	0	1,803	0.36 (0.25)	Ref.	<0.001		Ref.			
		4	1,681	0.26 (0.19)	-29.4%	<0.001		-0.11 (-0.12,-0.09)	<0.001	0.070	
		6	1,624	0.29 (0.24)	-18.6%	<0.001		-0.07 (-0.08,-0.05)	<0.001	0.009	
		8	1,704	0.30 (0.25)	-15.8%	<0.001		-0.06 (-0.07,-0.04)	<0.001	0.003	
		10	1,803	0.30 (0.22)	-18.4%	<0.001		-0.07 (-0.08,-0.05)	<0.001	0.033	
	T2D-p	0	183	0.29 (0.28)	Ref.	<0.001	<0.001	Ref.			
		4	171	0.16 (0.19)	-44.5%	<0.001	<0.001	-0.13 (-0.17,-0.08)	<0.001	0.089	0.390
		6	169	0.18 (0.20)	-38.6%	<0.001	<0.001	-0.11 (-0.16,-0.07)	<0.001	0.002	0.054
		8	164	0.19 (0.21)	-36.2%	<0.001	<0.001	-0.11 (-0.15,-0.06)	<0.001	0.001	0.064
		10	183	0.16 (0.18)	-43.6%	<0.001	<0.001	-0.13 (-0.17,-0.08)	<0.001	<0.001	0.010

CI, confidence interval; SD, standard deviation; mSI, model-derived sensitivity of insulin; mBCF, model-derived beta-cell function; Non-p, nonprogressor; PreDM-p, progressor to prediabetes; T2D-p, progressor to type 2 diabetes.

¶ P-value derived from paired t-tests comparing each time point with baseline (time = 0) within the same group.

† P-value derived from independent t-tests comparing the Non-p group with other subgroups.

‡ P-value derived from independent t-tests comparing the preDM-p group with other subgroups.

Supplemental Table S2. Longitudinal Increments of body mass index (BMI) and Fat Mass (Details of Fig. 1C and D)

			Raw data					Change from baseline			
						Comparison between groups				Comparison between groups	
	Subgroup	Time (year)	N	Mean (SD)	Percentage change	p-value†	p-value‡	Mean difference (95%CI)	p-value ¶	p-value†	p-value‡
BMI	Non-p	0	824	23.57 (2.77)	Ref.			Ref.			
		4	741	23.50 (2.70)	-0.3%			-0.06 (-0.15,0.02)	0.149		
		6	722	23.42 (2.75)	-0.6%			-0.13 (-0.23,-0.04)	0.008		
		8	745	23.46 (2.74)	-0.4%			-0.09 (-0.19,0.01)	0.076		
		10	824	23.54 (2.83)	-0.1%			-0.03 (-0.14,0.07)	0.515		
	PreDM-p	0	1,802	24.39 (2.87)	Ref.	<0.001		Ref.			
		4	1,680	24.38 (2.88)	-0.1%	<0.001		-0.01 (-0.07,0.04)	0.654	0.340	
		6	1,624	24.38 (2.84)	0.0%	<0.001		-0.01 (-0.07,0.05)	0.758	0.038	
		8	1,702	24.39 (2.92)	0.0%	<0.001		-0.00 (-0.07,0.07)	0.995	0.140	
		10	1,801	24.50 (3.02)	0.4%	<0.001		0.11 (0.04,0.18)	0.002	0.024	
	T2D-p	0	182	24.25 (3.06)	Ref.	0.006	0.550	Ref.			
		4	170	24.24 (3.25)	0.2%	0.006	0.580	0.06 (-0.13,0.24)	0.565	0.260	0.500
		6	168	24.49 (3.30)	0.6%	<0.001	0.670	0.14 (-0.07,0.35)	0.181	0.020	0.170
		8	163	24.41 (3.41)	0.8%	0.001	0.950	0.18 (-0.05,0.41)	0.118	0.033	0.130
		10	182	24.51 (3.56)	1.1%	<0.001	0.960	0.26 (0.03,0.50)	0.029	0.024	0.220
Body Fat Free Mass (kg)	Non-p	0	625	15.29 (4.72)	Ref.			Ref.			
		4	557	14.72 (4.57)	-3.2%			-0.48 (-0.68,-0.29)	<0.001		
		6	538	14.89 (4.72)	-2.3%			-0.35 (-0.58,-0.11)	0.004		
		8	554	15.00 (4.64)	-1.3%			-0.20 (-0.42,0.02)	0.069		

			Raw data					Change from baseline			
						Comparison between groups				Comparison between groups	
	Subgroup	Time (year)	N	Mean (SD)	Percentage change	p-value†	p-value‡	Mean difference (95%CI)	p-value ¶	p-value†	p-value‡
		10	621	15.39 (4.90)	0.7%			0.10 (-0.12,0.33)	0.367		
	PreDM-p	0	1,400	16.70 (5.02)	Ref.	<0.001		Ref.			
		4	1,291	16.46 (5.01)	-1.3%	<0.001		-0.22 (-0.35,-0.08)	0.002	0.030	
		6	1,264	16.74 (5.12)	0.0%	<0.001		0.01 (-0.15,0.16)	0.943	0.013	
		8	1,322	16.76 (5.14)	0.4%	<0.001		0.06 (-0.09,0.22)	0.429	0.053	
		10	1,396	17.05 (5.27)	2.1%	<0.001		0.34 (0.18,0.50)	<0.001	0.094	
	T2D-p	0	148	16.15 (4.89)	Ref.	0.054	0.200	Ref.			
		4	141	16.26 (5.41)	1.4%	0.002	0.680	0.22 (-0.19,0.63)	0.293	0.003	0.047
		6	138	16.54 (5.40)	1.8%	0.001	0.680	0.30 (-0.14,0.74)	0.186	0.012	0.220
		8	133	16.22 (5.33)	0.7%	0.016	0.260	0.12 (-0.35,0.59)	0.617	0.220	0.820
		10	148	16.67 (5.69)	3.2%	0.013	0.440	0.52 (0.01,1.03)	0.049	0.150	0.520

CI, confidence interval; SD, standard deviation; BMI, body mass index, Non-p, nonprogressor; PreDM-p, progressor to prediabetes; T2D-p, progressor to type 2 diabetes
¶ P-value derived from paired t-tests comparing each time point with baseline (time = 0) within the same group.
† P-value derived from independent t-tests comparing the Non-p group with other subgroups.
‡ P-value derived from independent t-tests comparing the preDM-p group with other subgroups.

Supplemental Table S3. Longitudinal Changes of Matsuda and IGI (Details of Supplemental Fig. 3)

			Raw data					Change from baseline			
						Comparison between groups				Comparison between groups	
	Subgroup	Time (year)	N	Mean (SD)	Percentage change	p-value†	p-value‡	Mean difference (95%CI)	p-value ¶	p-value†	p-value‡
Matsuda	Non-p	0	824	14.13 (10.64)	Ref.			Ref.			
		4	742	12.47 (5.41)	-11.9%			-1.68 (-2.49,-0.88)	<0.001		
		6	722	10.48 (6.81)	-26.5%			-3.78 (-4.61,-2.95)	<0.001		
		8	745	9.92 (5.39)	-29.3%			-4.11 (-4.93,-3.29)	<0.001		
		10	824	10.73 (6.08)	-24.1%			-3.40 (-4.17,-2.63)	<0.001		
	PreDM-p	0	1,803	12.27 (9.03)	Ref.	<0.001		Ref.			
		4	1,681	10.15 (5.13)	-16.6%	<0.001		-2.02 (-2.47,-1.58)	<0.001	0.470	
		6	1,624	8.00 (4.21)	-34.4%	<0.001		-4.20 (-4.64,-3.76)	<0.001	0.380	
		8	1,704	7.43 (4.16)	-39.7%	<0.001		-4.89 (-5.34,-4.44)	<0.001	0.100	
		10	1,803	8.02 (4.66)	-34.7%	<0.001		-4.26 (-4.68,-3.84)	<0.001	0.056	
	T2D-p	0	183	11.82 (7.76)	Ref.	<0.001	0.460	Ref.			
		4	171	8.73 (5.41)	-26.6%	<0.001	0.001	-3.16 (-4.42,-1.89)	<0.001	0.056	0.099
		6	169	6.10 (3.87)	-48.3%	<0.001	<0.001	-5.70 (-6.89,-4.50)	<0.001	0.010	0.022
		8	164	5.95 (3.56)	-51.0%	<0.001	<0.001	-6.20 (-7.41,-4.98)	<0.001	0.006	0.049
		10	183	6.17 (3.87)	-47.8%	<0.001	<0.001	-5.65 (-6.77,-4.53)	<0.001	0.001	0.023
IGI	Non-p	0	652	1.13 (3.20)	Ref.			Ref.			
		4	494	0.96 (2.00)	-15.2%			-0.17 (-0.53,0.18)	0.341		
		6	490	1.27 (3.11)	6.3%			0.08 (-0.34,0.49)	0.722		
		8	519	1.51 (2.51)	50.0%			0.50 (0.28,0.73)	<0.001		

			Raw data					Change from baseline			
						Comparison between groups				Comparison between groups	
	Subgroup	Time (year)	N	Mean (SD)	Percentage change	p-value†	p-value‡	Mean difference (95%CI)	p-value ¶	p-value†	p-value‡
		10	571	1.51 (4.26)	35.3%			0.39 (0.02,0.77)	0.038		
	PreDM-p	0	1,485	0.99 (3.11)	Ref.	0.350		Ref.			
	PreDM-p	4	1,223	0.71 (1.24)	-22.5%	0.008		-0.20 (-0.32,-0.09)	<0.001	0.870	
	PreDM-p	6	1,219	0.96 (1.78)	1.8%	0.039		0.02 (-0.19,0.22)	0.872	0.800	
	PreDM-p	8	1,304	0.98 (1.54)	0.9%	<0.001		0.01 (-0.18,0.20)	0.923	0.001	
	PreDM-p	10	1,398	0.97 (2.03)	0.8%	0.004		0.01 (-0.18,0.19)	0.936	0.068	
	T2D-p	0	151	0.60 (0.81)	Ref.	<0.001	<0.001	Ref.			
	T2D-p	4	129	0.47 (0.62)	-15.3%	<0.001	<0.001	-0.08 (-0.23,0.06)	0.263	0.650	0.210
	T2D-p	6	130	0.54 (0.69)	-14.1%	<0.001	<0.001	-0.09 (-0.26,0.08)	0.316	0.470	0.440
	T2D-p	8	126	0.52 (0.54)	-16.4%	<0.001	<0.001	-0.10 (-0.26,0.05)	0.196	<0.001	0.370
	T2D-p	10	150	0.52 (0.73)	-12.7%	<0.001	<0.001	-0.08 (-0.23,0.08)	0.341	0.022	0.500

CI, confidence interval; SD, standard deviation; IGI, insulinogenic index Non-p, nonprogressor; PreDM-p, progressor to prediabetes; T2D-p, progressor to type 2 diabetes.

¶ P-value derived from paired t-tests comparing each time point with baseline (time = 0) within the same group.

† P-value derived from independent t-tests comparing the Non-p group with other subgroups.

‡ P-value derived from independent t-tests comparing the preDM-p group with other subgroups.

Supplemental Table S4. Longitudinal Changes of oral disposition index (oDI) and model-derived disposition index (mDI) (Details of Supplemental Fig. 4)

			Raw data					Change from baseline			
						Comparison between groups				Comparison between groups	
	Subgroup	Time (year)	N	Mean (SD)	Percentage change	p-value†	p-value‡	Mean difference (95%CI)	p-value ¶	p-value†	p-value‡
oDI	Non-p	0	652	13.09 (36.93)	Ref.			Ref.			
		4	494	9.86 (22.34)	-23.0%			-2.95 (-6.98,1.07)	0.151		
		6	490	10.47 (24.11)	-22.3%			-3.00 (-7.20,1.20)	0.162		
		8	519	11.02 (17.45)	-2.4%			-0.27 (-2.37,1.84)	0.804		
		10	571	13.00 (32.24)	0.5%			0.07 (-3.28,3.42)	0.968		
	PreDM-p	0	1,485	9.42 (26.15)	Ref.	0.022		Ref.			
		4	1,223	5.95 (13.81)	-31.7%	<0.001		-2.75 (-4.06,-1.45)	<0.001	0.930	
		6	1,219	6.67 (15.09)	-23.4%	0.001		-2.04 (-3.61,-0.47)	0.011	0.680	
		8	1,304	6.26 (13.05)	-32.7%	<0.001		-3.04 (-4.64,-1.44)	<0.001	0.040	
		10	1,398	6.53 (14.29)	-27.1%	<0.001		-2.43 (-3.91,-0.94)	0.001	0.180	
	T2D-p	0	151	5.58 (7.59)	Ref.	<0.001	<0.001	Ref.			
		4	129	3.27 (6.61)	-36.2%	<0.001	<0.001	-1.85 (-3.31,-0.40)	0.014	0.620	0.370
		6	130	2.82 (4.14)	-50.7%	<0.001	<0.001	-2.90 (-4.34,-1.46)	<0.001	0.970	0.430
		8	126	2.83 (3.38)	-52.3%	<0.001	<0.001	-3.11 (-4.46,-1.75)	<0.001	0.027	0.950
		10	150	2.93 (5.20)	-47.2%	<0.001	<0.001	-2.62 (-3.93,-1.32)	<0.001	0.140	0.840
mDI	Non-p	0	824	3.76 (1.68)	Ref.			Ref.			
		4	742	3.21 (1.69)	-14.8%			-0.56 (-0.70,-0.42)	<0.001		
		6	722	3.18 (1.68)	-16.7%			-0.64 (-0.79,-0.49)	<0.001		
		8	745	2.96 (1.64)	-21.8%			-0.83 (-0.97,-0.69)	<0.001		

			Raw data					Change from baseline			
						Comparison between groups				Comparison between groups	
	Subgroup	Time (year)	N	Mean (SD)	Percentage change	p-value†	p-value‡	Mean difference (95%CI)	p-value ¶	p-value†	p-value‡
		10	824	2.93 (1.61)	-22.2%			-0.83 (-0.97,-0.70)	<0.001		
	PreDM-p	0	1,803	2.79 (1.55)	Ref.	<0.001		Ref.			
		4	1,681	1.93 (1.47)	-31.0%	<0.001		-0.87 (-0.95,-0.79)	<0.001	<0.001	
		6	1,624	1.76 (1.40)	-37.1%	<0.001		-1.04 (-1.12,-0.96)	<0.001	<0.001	
		8	1,704	1.58 (1.32)	-43.4%	<0.001		-1.21 (-1.29,-1.14)	<0.001	<0.001	
		10	1,803	1.64 (1.31)	-41.4%	<0.001		-1.16 (-1.23,-1.08)	<0.001	<0.001	
	T2D-p	0	183	2.02 (1.37)	Ref.	<0.001	<0.001	Ref.			
		4	171	0.96 (1.30)	-52.6%	<0.001	<0.001	-1.07 (-1.29,-0.84)	<0.001	<0.001	0.099
		6	169	0.76 (0.97)	-63.2%	<0.001	<0.001	-1.30 (-1.51,-1.09)	<0.001	<0.001	0.026
		8	164	0.77 (1.12)	-62.7%	<0.001	<0.001	-1.29 (-1.51,-1.08)	<0.001	<0.001	0.510
		10	183	0.68 (0.94)	-66.4%	<0.001	<0.001	-1.34 (-1.53,-1.16)	<0.001	<0.001	0.069

CI, confidence interval; SD, standard deviation; oDI, oral disposition index; mDI, model-derived disposition index; Non-p, nonprogressor; PreDM-p, progressor to prediabetes; T2D-p, progressor to type 2 diabetes.

¶ P-value derived from paired t-tests comparing each time point with baseline (time = 0) within the same group.

† P-value derived from independent t-tests comparing the Non-p group with other subgroups.

‡ P-value derived from independent t-tests comparing the preDM-p group with other subgroups.

Supplemental Table S5. Multiple logistic regression analyses for the effect of decreased insulin sensitivity on progression to prediabetes and type 2 diabetes

Model	Progression to PreDM		Progression to T2D	
	OR 95% Cl	*P*-value	OR 95% Cl	*P*-value
1	1.33 [1.17, 1.51]	<0.001	1.87 [1.47, 2.38]	<0.001
2	1.36 [1.19, 1.55]	<0.001	1.96 [1.54, 2.51]	<0.001
3	1.89 [1.60, 2.23]	<0.001	4.52 [3.15, 6.49]	<0.001
4	1.77 [1.50, 2.09]	<0.001	3.99 [2.76, 5.87]	<0.001
5	1.91 [1.61, 2.27]	<0.001	4.84 [3.31, 7.06]	<0.001

Model 1, crude (DIS); model 2, adjusted for age, sex and BMI; model 3, adjusted for model 2 + baseline IS; model 4, adjusted for model 3 + baseline BCF; model 5, adjusted for model 4 + DBCF

DIS: Decreased insulin sensitivity = insulin sensitivity at year 0 - insulin sensitivity at year 10

DBCF: Decreased beta-cell function = BCF at year 0 - BCF at year 10

OR, odd ratio; CI, confidence interval; PreDM, prediabetes, T2D; type 2 diabetes; BMI, body mass index; IS, insulin sensitivity; BCF, beta-cell function.

Supplemental Table S6. Incidence of diabetes and pre-diabetes in groups of high and low insulin sensitivity, subdivided by small and large decrease in beta-cell function (DBCF)

	Low insulin sensitivity			High insulin sensitivity		
	Large DBCF (N=703)	small DBCF (N=702)	*P* value	Large DBCF (N=703)	small DBCF (N=702)	*P* value
T2D-p	39 (5.5%)	16 (2.3%)	0.002	23 (3.3%)	12 (1.7%)	0.060
PreDM-p	313 (44.5%)	252 (35.9%)	0.001	288 (41.0%)	201 (28.6%)	<0.001
Non-p	351 (42.7%)	434 (61.8%)	<0.001	392 (55.8%)	489 (69.7%)	<0.001

DBCF: Decreased beta-cell function = beta-cell function at year 0 – beta-cell function at year 10

Supplemental Fig. S1. Flow chart for the study participants

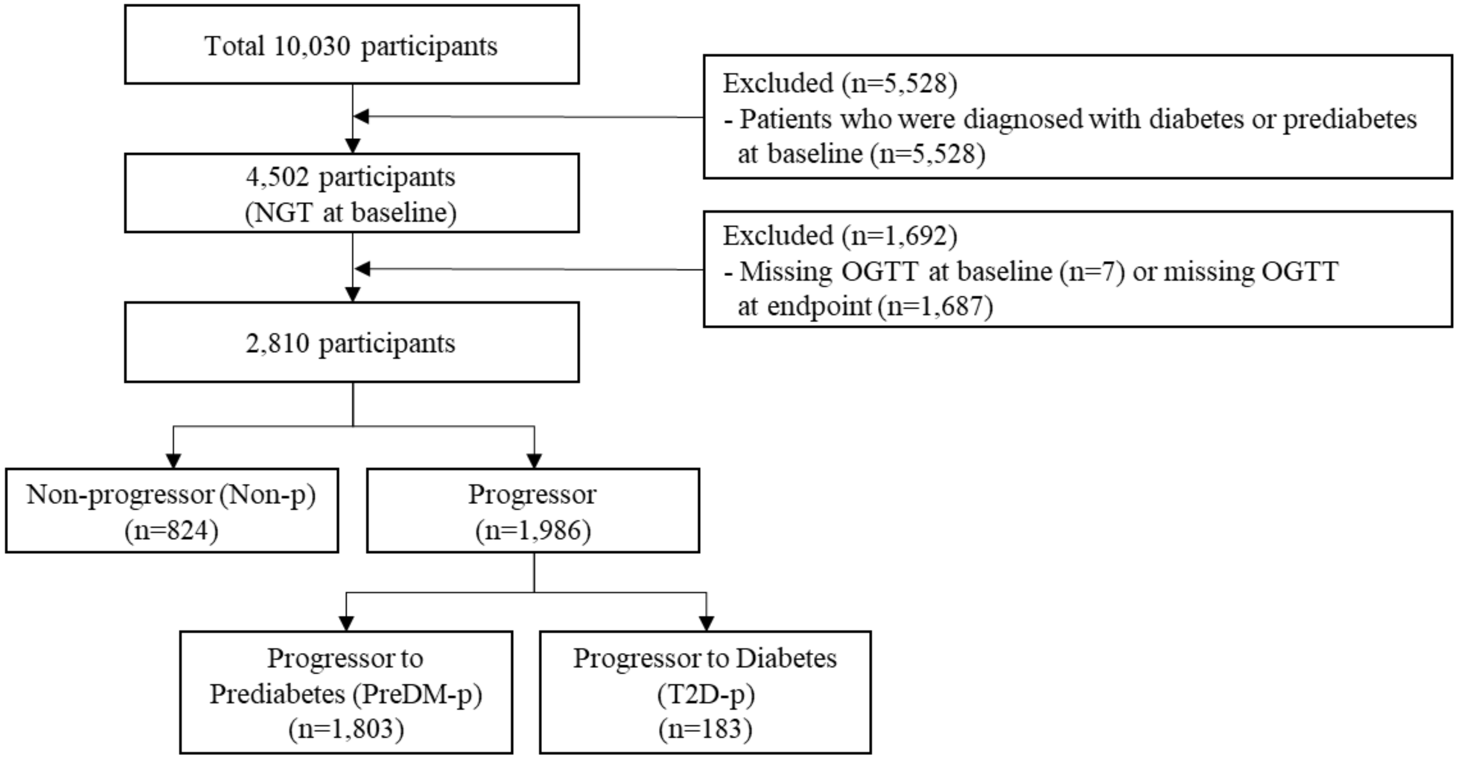

Supplementary Fig. 1. Flow chart for selection of population. Normal glucose tolerance (NGT), oral glucose tolerance test (OGTT)

Supplemental Fig. S2. Worsening insulin resistance leads to T2D: Longitudinal simulations of a mathematical model

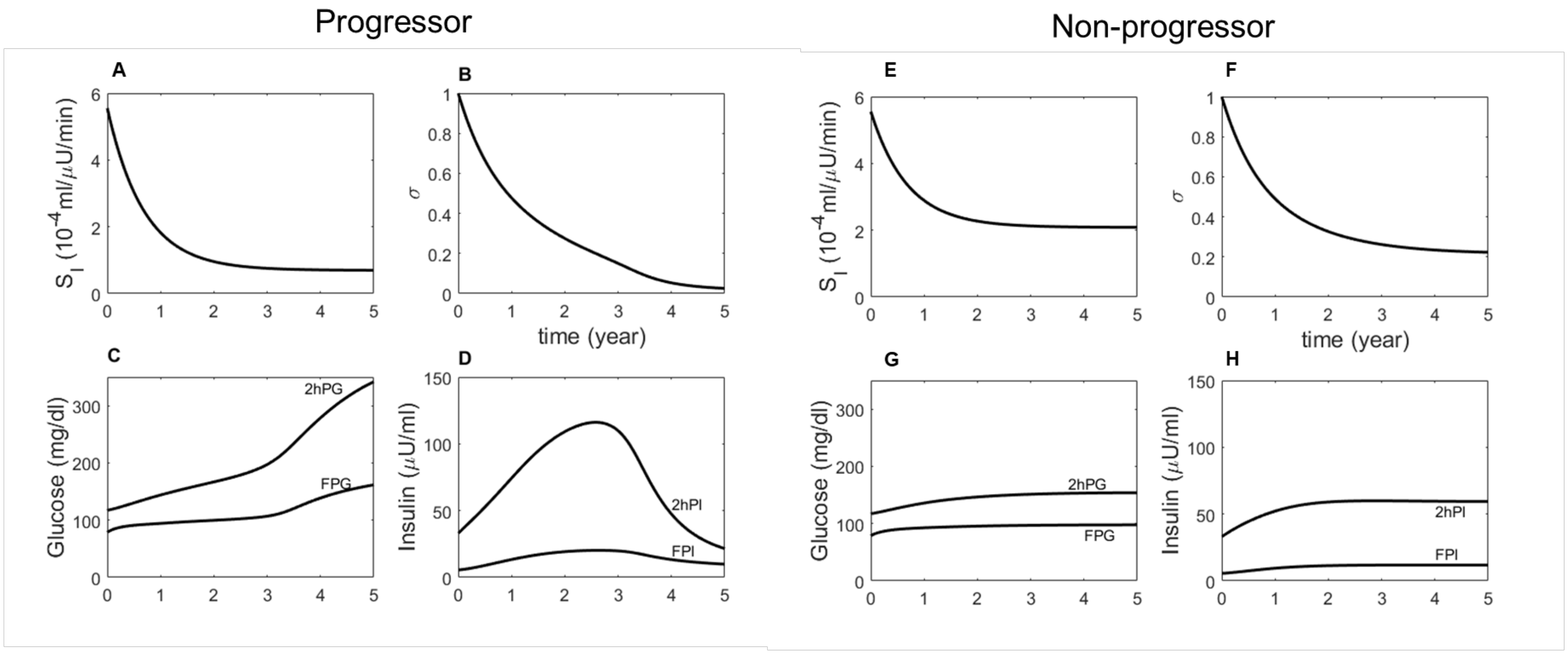

Supplementary Fig. 2. Worsening insulin resistance leads to T2D: Longitudinal simulations of a mathematical model. Assume beta-cell function of the progressor and non-progressor are the same at baseline. (A-D) Simulations of a progressor. A) Assume that insulin sensitivity decreases over time, more than than the non-progressor (E). B) Beta-cell function (σ) declines progressively throughout, worser than the non-progressor (F). C) Simulated fasting plasma glucose (FPG) and 2-hour glucose (2hPG) progress over time, where FPG and 2hPG were obtained when OGTTs are simulated every week. D) Simulated fasting plasma insulin (FPI) and 2-hour insulin (2hPI) initially increase and decline, resulting from decompensation. (E-H) Simulation of a non-progressor. E) Assume that insulin sensitivity decreases moderately over time. F) Beta-cell function (σ) decreases first and does not get worse. G) Simulated FPG and 2hPG slightly increase and saturate. H) Simulated FPI and 2hPI increase and saturates but never declines.

Supplemental Fig. S3. Longitudinal changes of Matsuda index and IGI

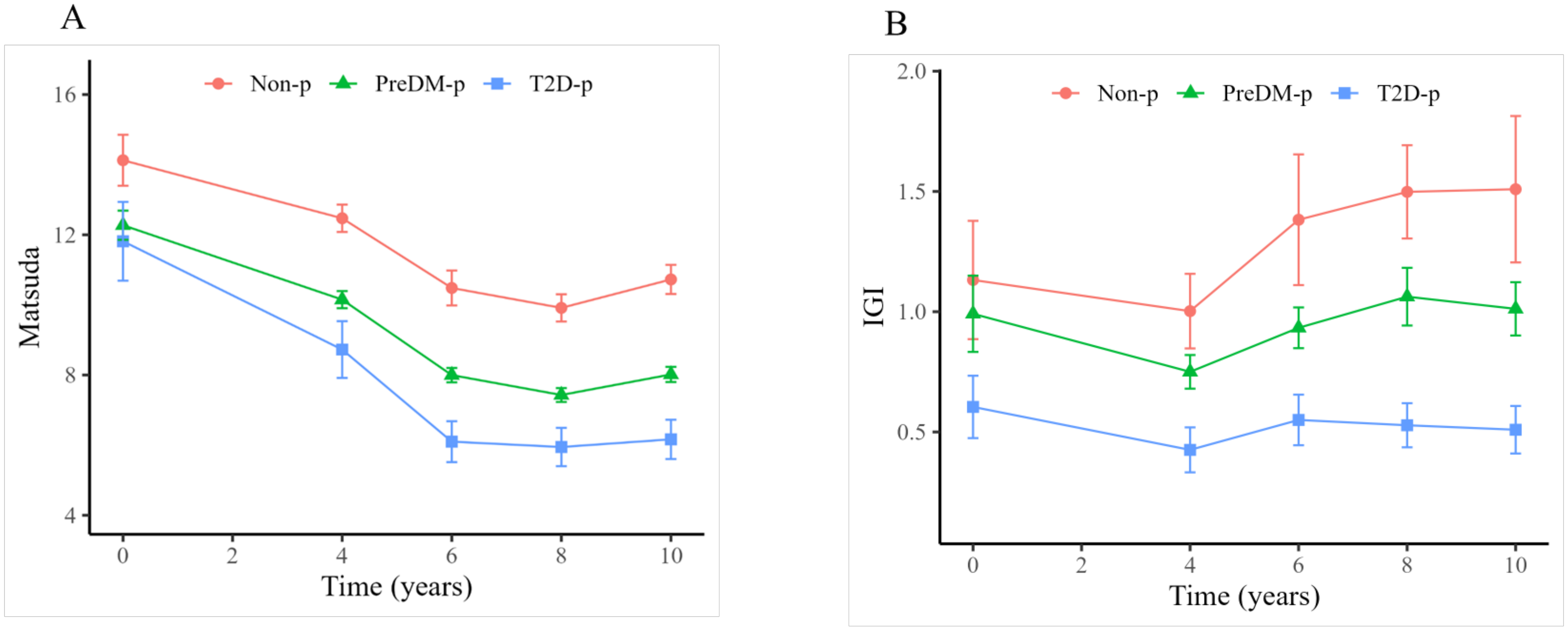

Supplementary Fig. 3. Longitudinal changes of Matsuda index and insulinogenic index (IGI). (A) At baseline, Matsuda is higher in the non-progressors (Non-p) than the two progressor groups, but there is no difference in Matsuda index between the progressors to prediabetes (PreDM-p) and the progressors to type 2 diabetes (T2D-p). Over time, Matsuda index is decreased in all groups. T2D-p group decrease most, PreDM-p group intermediate, and Non-p group the least. (B) IGIs are different between the three groups at baseline (T2D-p vs. PreDM-p and Non-p, all $P<0.001$) and the two progressor groups stay low over time. See supplemental Table S3 for detailed statistics.

Supplemental Fig. S4. Longitudinal changes of oDI and mDI

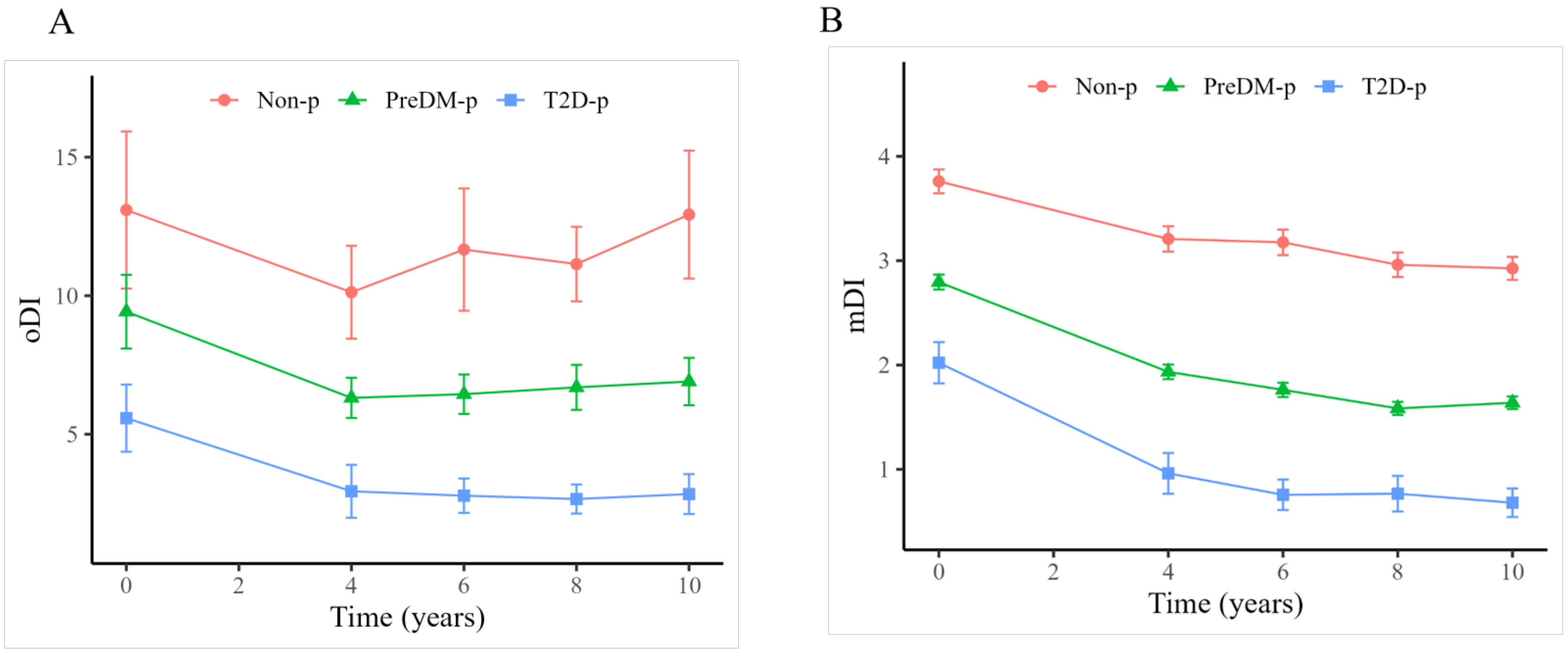

Supplementary Fig. 4. Longitudinal changes of oral disposition index (oDI) and model-derived disposition index (mDI). (A) At baseline, oDI is higher in non-progressors than the two progressor groups (both $P<0.05$). oDI is decreased in PreDM progressor groups and T2DM progressor groups (both $P<0.05$ compared with each baseline). (B) At baseline, mDI is higher in non-progressors than the two progressor groups (both $P<0.05$). mDI decrease over time in all three groups (both $P<0.05$ compared with each baseline). Supplemental Table S4 for detailed statistics.
